# Preventive effect of goshajinkigan on paclitaxel-induced peripheral neuropathy: an open-label, randomized phase II study (OLCSG2101)

**DOI:** 10.1093/oncolo/oyag352

**Published:** 2026-09-16

**Authors:** Takaaki Tanaka, Go Makimoto, Naoki Nakamura, Yuka Kato, Isao Oze, Toshihide Yokoyama, Shoichi Kuyama, Satoru Senoo, Koji Inoue, Hirohisa Ichikawa, Yoshinobu Maeda, Yosuke Togashi, Katsuyuki Hotta

**Affiliations:** Department of Hematology, Oncology and Respiratory Medicine, Graduate School of Medicine, Dentistry and Pharmaceutical Sciences, Okayama University, Okayama 700-8558, Japan; Department of Respiratory Medicine, Okayama University Hospital, Okayama 700-8558, Japan; Department of Hematology, Oncology and Respiratory Medicine, Graduate School of Medicine, Dentistry and Pharmaceutical Sciences, Okayama University, Okayama 700-8558, Japan; Department of Thoracic Oncology and Medicine, National Hospital Organization Shikoku Cancer Center, Ehime 791-0280, Japan; Division of Cancer Information and Control, Aichi Cancer Center Research Institute, Nagoya 464-8681, Japan; Department of Respiratory Medicine, Kurashiki Central Hospital, Okayama 710-8602, Japan; Department of Respiratory Medicine, Iwakuni Clinical Center, Yamaguchi 740-8510, Japan; Department of Respiratory Medicine, NHO Fukuyama Medical Center, Hiroshima 720-8520, Japan; Department of Respiratory Medicine, Ehime Prefectural Central Hospital, Ehime 790-0024, Japan; Department of Respiratory Medicine, KKR Takamatsu Hospital, Kagawa 760-0018, Japan; Department of Respiratory Medicine, Okayama University Hospital, Okayama 700-8558, Japan; Department of Respiratory Medicine, Okayama University Hospital, Okayama 700-8558, Japan; Department of Tumor Microenvironment, Graduate School of Medicine, Dentistry and Pharmaceutical Sciences, Okayama University, Okayama 700-8558, Japan; Advanced Research Field, Institute for Emerging Medical Innovation, Okayama University Hospital, Okayama 700-8558, Japan; Kindai University, Faculty of Medicine, Osaka 590-0197, Japan; Center for Innovative Clinical Medicine, Okayama University Hospital, Okayama 700-8558, Japan

**Keywords:** chemotherapy-induced peripheral neuropathy, paclitaxel, goshajinkigan, Kampo, randomized controlled trial

## Abstract

**Background:**

Chemotherapy-induced peripheral neuropathy (CIPN) is a dose-limiting toxicity of paclitaxel (PTX)-containing regimens, and no preventive strategy has been established. We evaluated goshajinkigan (GJG), a traditional Japanese herbal medicine (Kampo), for preventing CIPN during PTX-containing chemotherapy.

**Materials and Methods:**

In this open-label, randomized phase II trial across 7 institutions in Japan, patients with malignant tumors scheduled for ≥4 courses of carboplatin plus PTX (≥150 mg/m²) were randomized 1:1 to GJG or control, with minimization by cancer type and age. The primary endpoint was grade ≥2 CIPN incidence within 4 courses. The protocol-prespecified primary analysis used an unstratified 2-sided log-rank test (α = 0.10); a supportive stratified analysis accounted for the minimization factors.

**Results:**

Sixty-five patients were randomized (GJG, 32; control, 33); 52 (80.0%) had lung cancer. Grade ≥2 CIPN occurred in 28.1% (9/32) of the GJG arm vs 51.5% (17/33) of the control arm; the protocol-prespecified unstratified log-rank *P* value was .013, with a corresponding HR of 0.34 (90% CI, 0.16-0.72). In the stratified analysis accounting for cancer type and age, the estimate remained in favor of GJG but was attenuated and less precise (HR, 0.48; 90% CI, 0.22-1.02; stratified log-rank *P* = .101). Grade 3-5 adverse-event incidence was similar between arms.

**Conclusion:**

GJG was associated with fewer grade ≥2 CIPN events in this predominantly lung-cancer population. Given the open-label design and uncertainty in the stratified analysis, these findings represent a phase II preventive signal warranting confirmation in a double-blind, placebo-controlled phase III trial.

**Clinical trial registration number:**

Japan Registry of Clinical Trials (jRCTs061210047).

## Graphical Abstract

**Graphical Abstract:**
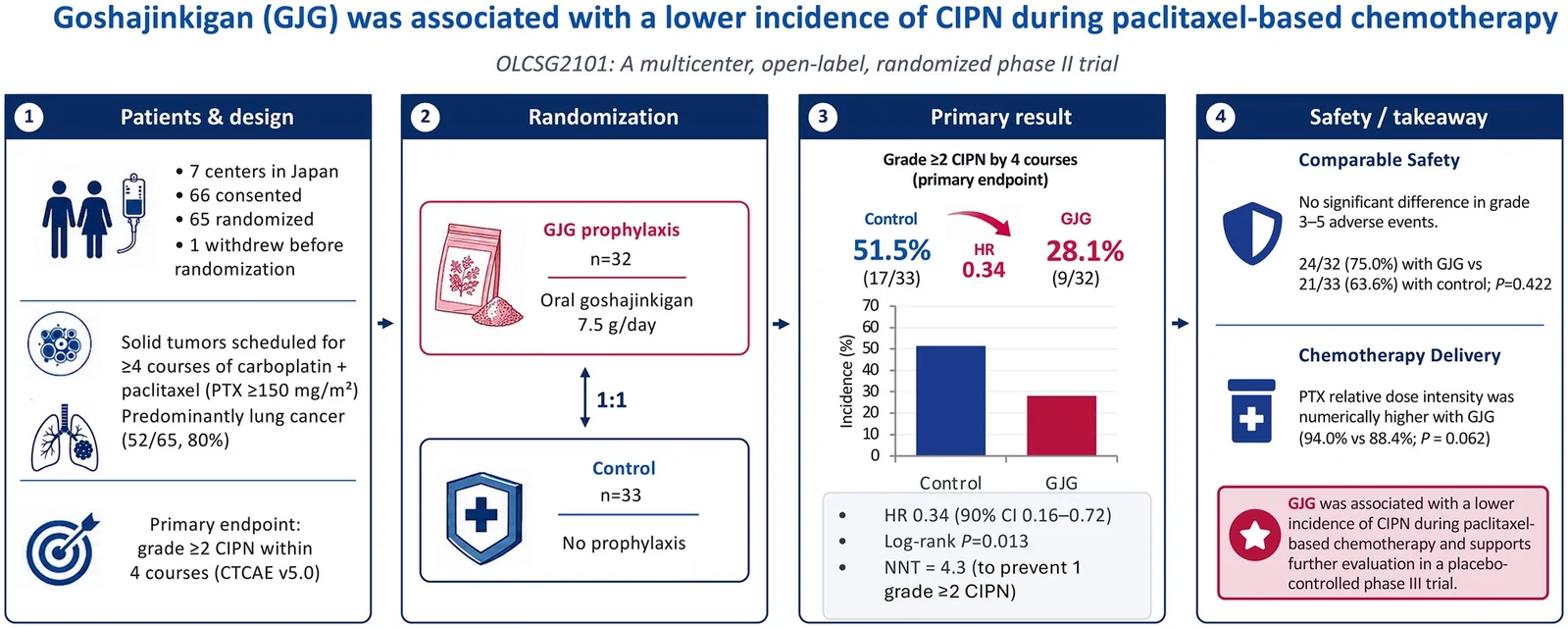

Implications for practice Paclitaxel-induced peripheral neuropathy can limit chemotherapy and impair quality of life, and no standard preventive drug has been established. In this small, open-label, randomized phase II trial of patients—mostly with lung cancer—receiving carboplatin plus paclitaxel, prophylactic goshajinkigan was associated with fewer grade ≥2 neuropathy events than no prophylaxis. In a stratified analysis accounting for the randomization factors, the estimate remained favorable but less precise, increasing uncertainty. Because the trial was small and unblinded and enrolled mostly patients with lung cancer, these results should not change current practice; they support a confirmatory double-blind, placebo-controlled phase III trial.

## Introduction

Paclitaxel (PTX) is a cornerstone of standard chemotherapy for multiple solid tumors, including advanced non-small-cell lung cancer (NSCLC), thymic carcinoma, and carcinoma of unknown primary. However, PTX frequently causes chemotherapy-induced peripheral neuropathy (CIPN); approximately 39% of patients with NSCLC receiving carboplatin plus PTX develop grade ≥2 peripheral sensory neuropathy.^1^ CIPN limits chemotherapy continuation and substantially impairs patients’ quality of life; nonetheless, the underlying pathophysiology remains incompletely understood.^2^

Management of established CIPN relies on delaying administration, reducing the dose, or discontinuing the offending agent. Duloxetine, pregabalin, and goshajinkigan (GJG) are used empirically for symptomatic relief, although supporting evidence remains limited.^2^^,^^3^ Major clinical practice guidelines do not endorse any pharmacologic intervention for CIPN prevention.^2^

GJG, a traditional Japanese herbal medicine (Kampo), has shown efficacy against diabetic neuropathy.^4^ Two systematic reviews and meta-analyses have evaluated GJG for CIPN prevention.^5^^,^^6^ The GENIUS trial—a double-blind, randomized phase III study of prophylactic GJG against oxaliplatin-induced CIPN in patients with colorectal cancer—paradoxically reported a higher incidence of CIPN in the GJG arm and was terminated early.^7^ However, CIPN induced by different anticancer agents involves pathologically distinct nerve damage: platinum compounds primarily affect the cell bodies, whereas taxanes cause axonal damage.^2^ A non-blinded randomized trial found that GJG significantly reduced docetaxel-induced CIPN compared with vitamin B12 in patients with breast cancer.^8^ GJG also suppressed PTX-induced mechanical allodynia in a rat model.^9^ A multicenter randomized trial compared prophylactic with delayed initiation of GJG for PTX-induced CIPN in patients with ovarian cancer^10^; the present study differs in population, comparator, endpoint definition, and design (see Discussion).

These findings suggest that GJG may be more effective against taxane-induced neuropathy than against platinum-induced neuropathy. We therefore conducted a randomized phase II trial (OLCSG2101) to determine whether prophylactic GJG can reduce the incidence of CIPN induced by PTX-containing chemotherapy. We selected carboplatin plus PTX as the index regimen because it is widely used across solid tumors, peripheral neuropathy is frequent with this combination, and platinum co-administration may add to cumulative neurotoxic exposure.^11^^,^^12^ Restricting eligibility to this single regimen also minimized between-patient heterogeneity. This trial was registered with the Japan Registry of Clinical Trials as OLCSG2101 (jRCTs061210047).^13^

## Methods

### Study design and oversight

We conducted a prospective, 2-arm, randomized, open-label phase II trial at 7 centers in Japan. The full study protocol has been published.^14^ A summary of the Certified Review Board (CRB)-approved protocol is provided as Supplementary Material; the full protocol document is available from the corresponding author upon reasonable request.

### Patients

Eligible patients were aged ≥20 years, had a pathologically confirmed malignant tumor and an Eastern Cooperative Oncology Group performance status of 0 or 1, and were scheduled to receive at least 4 courses of carboplatin plus PTX (PTX ≥150 mg/m², every 3-4 weeks). Concomitant administration of angiogenesis inhibitors (eg, bevacizumab) or immune checkpoint inhibitors (eg, atezolizumab, pembrolizumab) was permitted. We excluded patients with prior taxane exposure or preexisting peripheral neuropathy, and those receiving neuropathic pain medications such as duloxetine, pregabalin, or GJG.

### Randomization and treatment

Patients were randomized 1:1 to the GJG arm or the control arm using minimization by cancer type (lung vs non-lung cancer) and age (<70 vs ≥70 years). Patients in the GJG arm received oral GJG (TJ-107; Tsumura & Co., Tokyo, Japan). This prescription Kampo formulation is approved by the Japanese Ministry of Health, Labor and Welfare, covered by national health insurance, and manufactured under Good Manufacturing Practice standards as a dried extract of ten crude drugs in fixed proportions. Each 7.5 g of TJ-107 granules contains 4.5 g of a dried extract prepared from Rehmannia root 5.0 g, Achyranthes root 3.0 g, Cornus fruit 3.0 g, Dioscorea rhizome 3.0 g, Plantago seed 3.0 g, Alisma rhizome 3.0 g, Poria sclerotium 3.0 g, Moutan bark 3.0 g, Cinnamon bark 1.0 g, and powdered processed Aconite root 1.0 g; these values denote the quantities of crude drug used to manufacture the standard daily dose rather than the content of the final extract.^15^ GJG was administered at 7.5 g/day (3 sachets) in 2 or 3 divided doses from day 1 of cycle 1 through at least day 22 of cycle 4. Administration timing relative to meals was unrestricted. Eligibility was not restricted by the “Sho” (traditional Kampo constitutional assessment). Patients in the control arm received no prophylactic intervention for CIPN. However, once grade ≥2 CIPN developed, symptomatic therapies, including GJG, were permitted in both arms. The protocol permitted concurrent cryotherapy (limb cooling) and compression therapy (surgical gloves) during PTX infusion in both arms, preferably starting from cycle 1. The allocation sequence was generated with the minimization algorithm of INDICE Cloud, a web-based registration and randomization system.^16^ Site investigators enrolled patients and received treatment assignments at registration, ensuring allocation concealment until randomization. Because the treatment allocation was misinterpreted, one patient assigned to the control arm received prophylactic GJG and was analyzed in that arm according to the intention-to-treat (ITT) principle.

### Endpoints

The primary endpoint was the incidence of grade ≥2 CIPN within the first 4 chemotherapy courses, evaluated using the Common Terminology Criteria for Adverse Events (CTCAE) version 5.0. The treating investigators assessed the CIPN grade at every course. Grade ≥2 CIPN was also operationalized to include neuropathy requiring pharmacologic intervention or resulting in PTX dose reduction, discontinuation, or cycle delay. These treatment decisions were made by the treating investigators and were not governed by standardized protocol-defined criteria.

Prespecified secondary endpoints were (1) time from chemotherapy initiation to the onset of grade ≥2 CIPN; (2) CIPN incidence and severity at each course, assessed by CTCAE grade and the Patient Neurotoxicity Questionnaire (PNQ); (3) PTX relative dose intensity (RDI); (4) adverse events other than the primary CIPN endpoint, including grade 3-5 events; (5) the proportion of patients with grade ≥2 CIPN by course 4, excluding those who discontinued before course 4 for reasons other than CIPN; and (6) time to treatment failure (TTF), which was added via a protocol amendment registered on January 19, 2023, during enrollment.

### Statistical analysis

We based the sample size on a prior trial of prophylactic GJG in patients with breast cancer treated with docetaxel,^8^ which reported an incidence of grade ≥2 CIPN of 18.2% in the GJG arm and 48.1% in the control arm. Assuming an incidence of 50% without prophylaxis and 20% with GJG, we determined that 27 patients per group (54 in total) were required to achieve a statistical power of 75% at a 2-sided α level of 0.10. After accounting for an approximate 20% dropout rate for reasons other than CIPN, we set the target enrollment at 33 patients per group (66 in total).

The protocol-prespecified primary analysis compared the time to the first occurrence of grade ≥2 CIPN between the arms using the Kaplan–Meier method and an unstratified log-rank test in the ITT population (all randomized patients), as specified in the CRB-approved protocol. We used Cox proportional hazards models to estimate hazard ratios (HRs) with 90% CIs as supportive effect estimates. Confidence levels follow the trial design: estimates for the primary endpoint and for the analyses most closely related to it—the per-protocol analysis, the supportive stratified analysis, the protocol-aligned fixed-horizon analysis, and the post hoc analysis excluding patients who received cryotherapy—are reported with 90% CIs, corresponding to the 2-sided α of 0.10 prespecified for this phase II trial, whereas secondary and exploratory estimates, including time to treatment failure and the subgroup analyses, are reported with conventional 95% CIs. The confidence level is stated explicitly for all reported CIs. We evaluated the proportional hazards assumption using Schoenfeld residuals. In a protocol-aligned fixed-horizon analysis, we assessed the cumulative incidence within 4 courses and derived the absolute risk reduction, relative risk, and number needed to treat (NNT). We also performed a post hoc sensitivity analysis excluding patients who received cryotherapy during PTX infusion. Because randomization used minimization by cancer type and age, we performed a stratified log-rank test and stratified Cox regression accounting for these factors. Although this analysis was not prespecified in the protocol, it was considered an important supportive analysis because it directly accounted for the minimization factors and is therefore presented alongside the protocol-prespecified primary analysis. Results of the PNQ^17^ were summarized by course. We assessed physician–patient agreement regarding the 4-level CTCAE and PNQ grades (0-3) using overall percent agreement and Cohen’s weighted κ with quadratic weights. We aligned the CTCAE grades with the PNQ scale (A = 0, B = 1, C = 2, D = 3) for direct comparison; no patient experienced CTCAE grade 4 or 5 events, and none reported PNQ grade E. At the dichotomized grade ≥2 cutoff, we calculated the sensitivity and specificity of the physician assessment using the patient-reported PNQ as the reference.

Three patients who lacked an evaluable post-treatment CIPN assessment because of intercurrent adverse events were censored at 1 day after treatment initiation. Because this censoring may be informative, we performed post hoc fixed-horizon sensitivity analyses under alternative event assignments for the 3 censored patients: all censored patients were assumed to have experienced grade ≥2 CIPN, all were assumed to have remained event-free, and, in a conservative scenario most unfavorable to GJG, only the censored patient in the GJG arm was assumed to have experienced an event. Secondary, subgroup, and course-specific analyses were exploratory and not adjusted for multiplicity. We did not apply formal interim analyses or stopping rules. All *P* values are 2-sided. We performed all statistical analyses using Stata 18.0 (StataCorp).

### Ethics approval statement

The CRB of Okayama University approved the study (CRB21-005; September 28, 2021), and the trial was registered with the Japan Registry of Clinical Trials (jRCTs061210047; October 21, 2021). All procedures adhered to the Declaration of Helsinki and Japan’s Ethical Guidelines for Medical and Biological Research Involving Human Subjects. Written informed consent was obtained from all participants before enrollment.

## Results

### Patient enrollment and baseline characteristics

**Figure 1.**
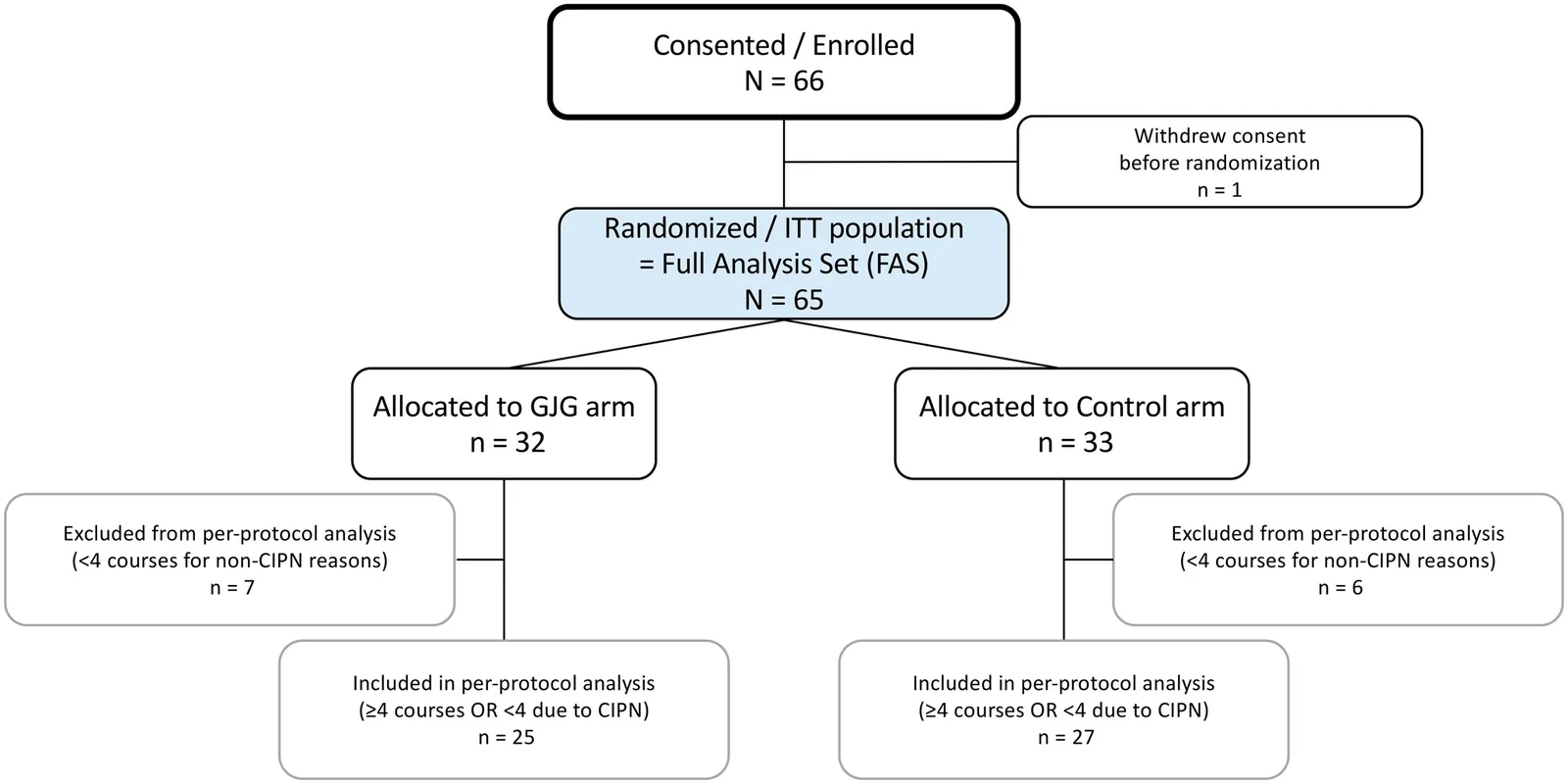
CONSORT flow diagram. Of 66 consented/enrolled patients, 1 withdrew consent before randomization and 65 were randomized to the GJG arm (*n* = 32) or the control arm (*n* = 33). The ITT population was identical to the FAS and included all randomized participants. The per-protocol population included patients who completed 4 chemotherapy courses or discontinued treatment before completing 4 courses because of CIPN; 13 patients were excluded for non-CIPN reasons before completing 4 courses. Three participants without evaluable post-treatment CIPN assessment were censored at 1 day after treatment initiation. The TTF analysis excluded 7 patients registered before the protocol amendment that added TTF as an endpoint. One patient in the control arm received GJG because of a misinterpretation of allocation and was analyzed in the control arm according to the ITT principle. Abbreviations: CIPN, chemotherapy-induced peripheral neuropathy; FAS, full analysis set; GJG, goshajinkigan; ITT, intention-to-treat; TTF, time to treatment failure.

Between April 2022 and September 2025, 66 patients were enrolled; 1 patient withdrew consent before randomization, leaving 65 patients in the ITT population (GJG arm, 32; control arm, 33; Figure 1). Baseline characteristics were well balanced between the 2 arms (Table 1). The median age was 68 years (IQR, 59-73). Most patients had lung cancer (52 of 65, 80.0%), and the most common regimen was carboplatin plus PTX with bevacizumab and atezolizumab (36 of 65, 55.4%). The median starting PTX dose was 191.6 mg/m² (IQR, 174.9-196.9), with a range of 151.9-202.6 mg/m^2^. For the per-protocol analysis, 52 patients were eligible (GJG arm, 25; control arm, 27); 13 patients were excluded because they discontinued treatment before completing 4 courses for reasons other than CIPN (GJG arm, 7; control arm, 6; Figure 1). The trial ended as planned upon reaching the accrual target.

**Table 1.** Baseline patient characteristics and concomitant CIPN-directed intervention (ITT population).

Characteristic	GJG arm (*n* = 32)	Control arm (*n* = 33)	Total (*N* = 65)
**Age, years, median [IQR]**	68.0 [56.5, 73.0]	68.0 [60.0, 74.0]	68.0 [59.0, 73.0]
**Sex, *n* (%)**			
** Male**	23 (71.9)	22 (66.7)	45 (69.2)
** Female**	9 (28.1)	11 (33.3)	20 (30.8)
**ECOG performance status, *n* (%)**			
** 0**	8 (25.0)	12 (36.4)	20 (30.8)
** 1**	24 (75.0)	21 (63.6)	45 (69.2)
**BMI, kg/m², median [IQR]**	22.1 [20.2, 23.5]	22.7 [21.1, 24.4]	22.3 [20.6, 24.0]
**Starting PTX dose, mg/m², median [IQR]**	194.1 [178.9, 199.2]	190.5 [171.1, 194.9]	191.6 [174.9, 196.9]
**Cancer type, *n* (%)**			
** Lung cancer (NSCLC)**	25 (78.1)	26 (78.8)	51 (78.5)
** Lung cancer (small cell)**	1 (3.1)	0 (0.0)	1 (1.5)
** Thymic cancer**	4 (12.5)	2 (6.1)	6 (9.2)
** Thymoma**	1 (3.1)	1 (3.0)	2 (3.1)
** Other^a^**	1 (3.1)	4 (12.1)	5 (7.7)
**Regimen, *n* (%)**			
** CBDCA + PTX + Bev + Atezo**	18 (56.2)	18 (54.5)	36 (55.4)
** CBDCA + PTX**	11 (34.4)	9 (27.3)	20 (30.8)
** CBDCA + PTX + Pembro**	2 (6.3)	4 (12.1)	6 (9.2)
** CBDCA + PTX + Bev**	1 (3.1)	2 (6.1)	3 (4.6)
**Treatment characteristics (post-randomization)**			
** Cryotherapy during PTX infusion^b^, *n* (%)**	6 (18.8)	2 (6.1)	8 (12.3)

Values are *n* (%) or median [IQR].

Abbreviations: Atezo, atezolizumab; Bev, bevacizumab; BMI, body mass index; CBDCA, carboplatin; CIPN, chemotherapy-induced peripheral neuropathy; ECOG PS, Eastern Cooperative Oncology Group performance status; GJG, goshajinkigan; IQR, interquartile range; ITT, intention-to-treat; NEC, neuroendocrine carcinoma; NSCLC, non-small cell lung cancer; Pembro, pembrolizumab; PTX, paclitaxel.

a Other includes NEC (*n* = 1), melanoma (*n* = 1), and carcinoma of unknown primary (*n* = 3).

b Cryotherapy (limb cooling) during PTX infusion was a concomitant intervention permitted by the protocol in both arms and was not a baseline characteristic; it is therefore reported separately as a post-randomization treatment characteristic. All 8 patients who received cryotherapy were treated at a single institution. No *P* value is reported for this comparison.

### Primary endpoint

**Figure 2.**
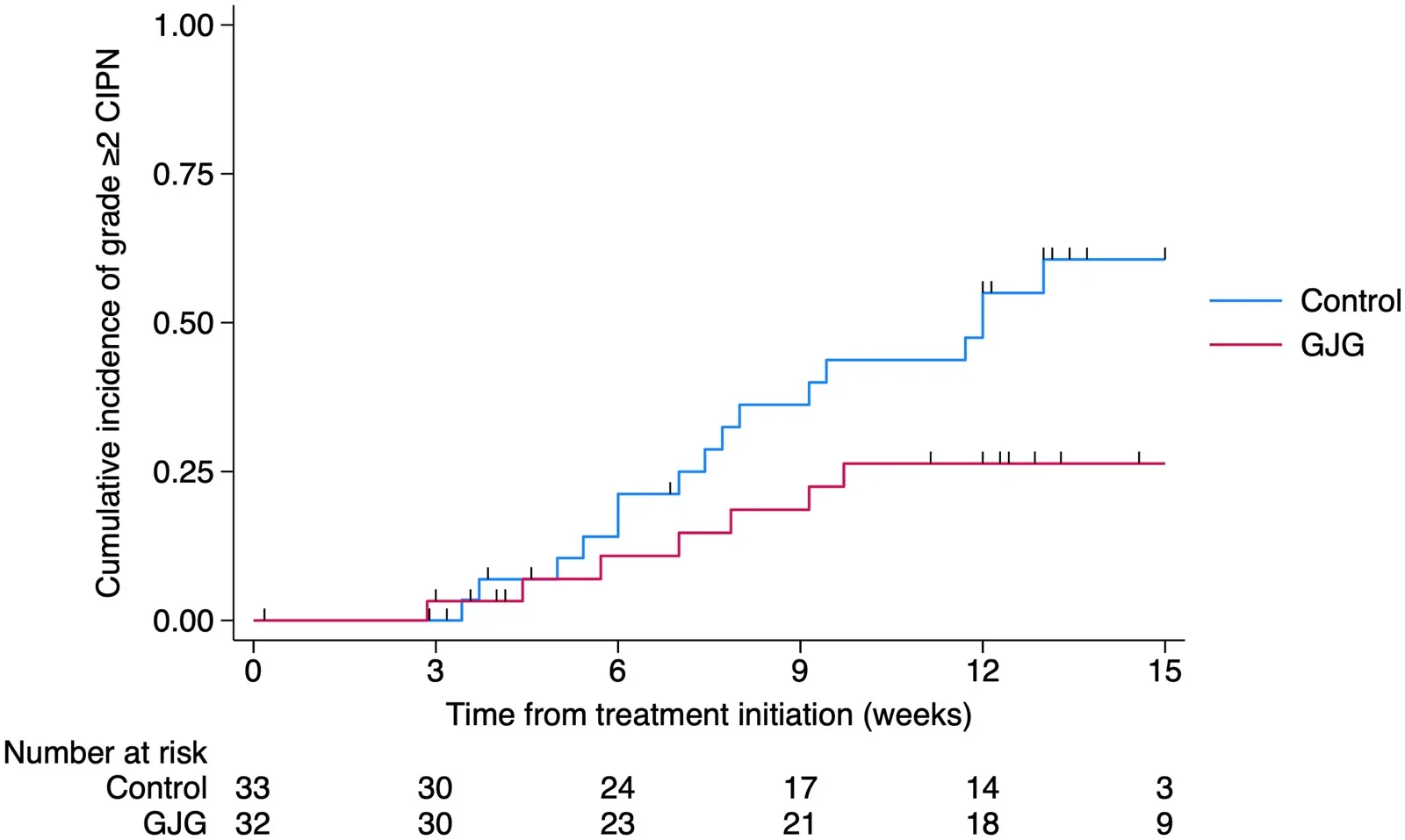
Kaplan-Meier curves for time to the first occurrence of grade ≥2 CIPN (intention-to-treat population). For visual clarity, the x-axis is truncated at 15 weeks (105 days). The numbers at risk are shown below the plot. The log-rank *P* value was .013 and was derived from the full follow-up data. Effect estimates with 90% CIs, corresponding to the 2-sided α = 0.10 prespecified for the phase II primary endpoint, are presented in Table 2. Abbreviations: CIPN, chemotherapy-induced peripheral neuropathy; GJG, goshajinkigan.

Grade ≥2 CIPN occurred in 9 of 32 patients (28.1%) in the GJG arm compared with 17 of 33 patients (51.5%) in the control arm (Table 2). In the protocol-prespecified primary time-to-event analysis, the unstratified log-rank *P* value was 0.013 (2-sided α  =  0.10; Table 2; Figure 2), and the corresponding unstratified HR was 0.34 (90% CI, 0.16-0.72; Table 2). In the stratified analysis accounting for the minimization factors (cancer type and age), the effect estimate remained in favor of GJG but was attenuated and less precise, with a 90% CI that narrowly included 1.0 (HR, 0.48; 90% CI, 0.22-1.02; stratified log-rank *P *= .101; Table 2). We found no evidence of a violation of the proportional hazards assumption (global *P *= .136). The supportive per-protocol analysis yielded consistent results (HR, 0.35; 90% CI, 0.17-0.75; log-rank *P *= .016; Table 2 and Supplementary Data 1). Supplementary Data 2 presents the per-course distribution of the CIPN grades, censored after the first grade ≥2 event. In the protocol-aligned fixed-horizon analysis, the absolute risk reduction (ARR) with GJG was 23.4 percentage points (90% CI, 4.0-42.8), corresponding to a relative risk of 0.55 (90% CI, 0.32-0.94) and an NNT of 4.3 (90% CI, 2.3-25.0) to prevent one grade ≥2 CIPN event within 4 courses (Table 2; Fisher exact *P *= .077).

**Table 2.** Primary endpoint analyses of grade ≥2 CIPN within 4 chemotherapy courses.

Analysis	Population/events	Effect estimate (90% CI)	*P* value
**Protocol-prespecified primary analysis (unstratified)**	ITT: GJG 9/32 (28.1%) vs control 17/33 (51.5%)	HR 0.34 (0.16-0.72)	.013
**Stratified analysis accounting for the minimization factors^a^**	ITT: GJG 9/32 (28.1%) vs control 17/33 (51.5%)	HR 0.48 (0.22-1.02)	.101
**Per-protocol analysis**	Per-protocol: GJG 9/25 (36.0%) vs control 17/27 (63.0%)	HR 0.35 (0.17-0.75)	.016
**Fixed-horizon analysis: absolute risk reduction (control − GJG)**	ITT: GJG 28.1% vs control 51.5%	23.4 pp (4.0-42.8)	.077^b^
**Fixed-horizon analysis: relative risk (GJG vs control)**	ITT: GJG 28.1% vs control 51.5%	0.55 (0.32-0.94)	—
**Fixed-horizon analysis: number needed to treat**	ITT	4.3 (2.3-25.0)	—

The protocol-prespecified primary comparison was the unstratified log-rank test; the corresponding HR was estimated using an unstratified Cox proportional hazards model. For the per-protocol analysis, the HR was estimated using an unstratified Cox model and the *P* value using an unstratified log-rank test.

Abbreviations: CIPN, chemotherapy-induced peripheral neuropathy; GJG, goshajinkigan; HR, hazard ratio; ITT, intention-to-treat; pp, percentage points.

a For the stratified analysis, the HR was estimated using stratified Cox regression and the *P* value using a stratified log-rank test, with cancer type and age group as strata. This analysis was not prespecified in the protocol and directly accounts for the minimization factors.

b Fisher exact test. All confidence intervals in this table are presented at the 90% level, corresponding to the 2-sided α = 0.10 prespecified for the phase II primary endpoint.

In post hoc fixed-horizon sensitivity analyses addressing the informative censoring of the 3 patients who discontinued treatment early, the direction of the effect remained unchanged. Under the assumption that all 3 censored patients experienced grade ≥2 CIPN, the risk was 31.3% in the GJG arm compared with 57.6% in the control arm (ARR, 26.3%; Fisher *P *= .046). Even in a conservative scenario most unfavorable to GJG, in which only the censored patient in the GJG arm was assumed to have experienced an event, the ARR remained 20.3% (Fisher *P *= .132). Cryotherapy during PTX infusion was used by 8 patients (GJG arm, 6 of 32; control arm, 2 of 33), all at a single institution. In a post hoc sensitivity analysis excluding these 8 patients (*n* = 57; 8 vs 15 events), the estimate remained in favor of GJG (HR, 0.42; 90% CI, 0.19-0.92; log-rank *P *= .061; Supplementary Data 3).

### Subgroup analysis

Supplementary Data 4 presents the prespecified exploratory subgroup analyses by the randomization factors. The HRs were 0.28 (95% CI, 0.09-0.87) for patients with lung cancer and 1.13 (95% CI, 0.25-5.11) for those with non-lung malignancies; by age, the HRs were 0.17 (95% CI, 0.04-0.81) for patients aged <70 years and 0.66 (95% CI, 0.21-2.01) for those aged ≥70 years. Interaction tests revealed no evidence of heterogeneity (*P *= .154 for cancer type and *P *= .193 for age), but did not establish its absence. All subgroups were small—only 13 patients had non-lung malignancies—and the corresponding CIs were wide. The trial was neither designed nor powered to test subgroup effects, and no threshold for interaction *P* values was prespecified; therefore, we cannot determine whether the differences in point estimates reflect true effect modification or chance imbalance.

### Time to treatment failure

We analyzed TTF in 58 patients after excluding the 7 patients registered before the protocol amendment that added TTF as an endpoint. Data for patients still receiving treatment were censored on January 31, 2026. TTF was similar between the 2 arms (GJG vs control: HR, 1.16; 95% CI, 0.70-1.94; *P *= .559; Supplementary Data 5 and 6).

### Safety

Grade 3-5 adverse events, regardless of attribution, occurred in 24 of 32 patients (75.0%) in the GJG arm and 21 of 33 patients (63.6%) in the control arm (*P *= .422; Table 3). Hematologic toxicity predominated, whereas non-hematologic grade 3-5 adverse events were similarly distributed between the arms (31.3% vs 27.3%; *P *= .789). No treatment-related deaths were reported, and no clear pattern of excess toxicity attributable to GJG was observed.

**Table 3.** Grade 3-5 adverse events (ITT population).

Event	GJG arm (*n* = 32) *n* (%)	Control arm (*n* = 33) *n* (%)	*P* ^a^
**Any grade 3-5 AE**	24 (75.0)	21 (63.6)	.422
**Hematologic**			
** Neutropenia**	18 (56.3)	18 (54.5)	1.000
** Febrile neutropenia**	4 (12.5)	4 (12.1)	1.000
** Thrombocytopenia/ITP**	0 (0.0)	2 (6.1)	.492
**Non-hematologic (any)**	10 (31.3)	9 (27.3)	.789
** Rash**	3 (9.4)	1 (3.0)	
** Anorexia**	1 (3.1)	3 (9.1)	
** Syncope**	0 (0.0)	3 (9.1)	
** Bacterial pneumonia**	1 (3.1)	1 (3.0)	
** Interstitial pneumonia**	1 (3.1)	0 (0.0)	
** Ileus**	1 (3.1)	0 (0.0)	
** Anaphylaxis**	1 (3.1)	0 (0.0)	
** Other^b^**	4 (12.5)	4 (12.1)	
**CIPN (grade 3)**	1 (3.1)	1 (3.0)	

Abbreviations: AE, adverse event; GJG, goshajinkigan; ITP, immune thrombocytopenia; ITT, intention-to-treat.

a Fisher exact test.

b GJG arm: fatigue, fever, fracture, swallowing dysfunction, hyponatremia, hyperkalemia (1 each). Control arm: hypertension, dehydration, encephalitis, hiccup (1 each). Other events are listed as event types; some patients had more than one event. Individual non-hematologic event categories are not mutually exclusive; a patient may contribute to more than one row. Adverse events are shown regardless of attribution to GJG or chemotherapy.

### Treatment delivery and adherence

The PTX RDI was numerically higher in the GJG arm (median, 94.0% vs 88.4%; *P *= .062; Table 4); per-course PTX doses are also shown in Table 4. GJG adherence data were not systematically collected in the case report form (CRF). However, no GJG-related treatment interruption or discontinuation was reported to the study secretariat during the protocol-defined administration period. Cryotherapy use is summarized in Table 1 (8 patients, all at a single institution); no patients received compression therapy.

**Table 4.** PTX relative dose intensity and per-course dosing (ITT population).

	GJG arm (*n* = 32)	Control arm (*n* = 33)	*P* ^a^
**PTX RDI, %, median (IQR)**	94.0 (89.0-98.1)	88.4 (85.3-96.3)	.062
**PTX RDI, %, mean (*SD*)**	92.9 (6.3)	89.6 (7.5)	
**Per-course PTX (mg/m²), mean (*SD*)**			
** Course 1**	189.7 (11.4)	182.5 (15.5)	.030
** Course 2**	182.4 (14.2)	180.3 (15.0)	.552
** Course 3**	184.3 (13.5)	175.2 (15.8)	.044
** Course 4**	183.2 (18.9)	174.2 (16.2)	.039

Abbreviations: BSA, body surface area; GJG, goshajinkigan; ITT, intention-to-treat; PTX, paclitaxel; RDI, relative dose intensity.

a Wilcoxon rank-sum test. RDI denominator: planned PTX 200 mg/m² × BSA × number of courses administered. Because the protocol permitted starting doses ≥150 mg/m² and the actual median starting dose was 191.6 mg/m², RDI values may underestimate the true proportion of intended dose delivered. This analysis should be considered exploratory.

### Physician-patient agreement in CIPN grading

Supplementary Data 7 presents the agreement between the physician-rated CIPN grade and the patient-reported PNQ. Exact agreement ranged from 57.6% (course 4) to 73.5% (course 2), with weighted kappa coefficients indicating moderate to substantial agreement (quadratic κ, 0.57-0.73). At the grade ≥2 cutoff, the sensitivity of the physician assessment declined from 66.7% at course 1 to 33.3% at course 4, whereas specificity remained high (81.8%-96.4%).

## Discussion

The present open-label, randomized phase II trial found that prophylactic GJG was associated with a lower occurrence of grade ≥2 CIPN during PTX-containing chemotherapy. A recent multicenter randomized trial evaluated GJG for PTX-induced CIPN in patients with ovarian cancer^10^; however, that study compared the prophylactic and delayed initiation of GJG and used the maximum sensory neuropathy grade and Visual Analog Scale as primary endpoints. In contrast, the present study enrolled mostly patients with lung cancer, permitted concomitant bevacizumab and immune checkpoint inhibitors, and compared upfront prophylactic GJG with no prophylaxis, with physician-rated CTCAE grade ≥2 CIPN within the first 4 courses as the primary endpoint. To our knowledge, this is the first randomized trial with a no-prophylaxis control group to evaluate prophylactic GJG for CIPN during PTX-containing chemotherapy.

The control-arm CIPN incidence (51.5%) was consistent with the assumed 50%, whereas the GJG-arm incidence (28.1%) slightly exceeded the assumed 20%. The effect size (ARR, 23.4 percentage points; NNT, 4.3) may be clinically meaningful. The relationship between the protocol-prespecified primary analysis and the stratified analysis accounting for the minimization factors warrants emphasis. Both estimates favor GJG (HR, 0.34 and 0.48, respectively), so the analyses differ in magnitude and precision rather than in direction; in the stratified analysis the estimate was attenuated and its 90% CI was wider and narrowly included 1.0. This leaves the precise magnitude and robustness of the treatment effect uncertain. The unstratified log-rank test nonetheless remains the protocol-prespecified primary analysis, and the result is best interpreted as a phase II preventive signal rather than as definitive evidence of efficacy.

The prespecified exploratory subgroup analyses showed no evidence of interaction by cancer type or age. The point estimate did not favor GJG in the non-lung cancer subgroup (HR, 1.13), whereas the estimate for patients aged ≥70 years remained numerically in favor of GJG (HR, 0.66); both estimates were imprecise. These analyses were not powered for definitive subgroup comparisons and should therefore be regarded as hypothesis-generating rather than as evidence of differential efficacy.

The distinct mechanisms of neuropathy induced by different agents may explain the difference in GJG efficacy between the present study and the GENIUS trial (oxaliplatin-induced CIPN).^7^ Platinum compounds primarily damage the dorsal root ganglion cell bodies, whereas taxanes cause axonal damage through microtubule stabilization.^2^ Our results are consistent with the prior randomized trial demonstrating GJG efficacy against docetaxel-induced CIPN in patients with breast cancer.^8^ GJG is a multicomponent extract, and no single crude drug has been established as responsible for its clinical effects, so the available mechanistic evidence relates to the mixture. Proposed mechanisms derive from preclinical work: in a rat model of PTX-induced neuropathy, GJG attenuated mechanical allodynia and was associated with less dorsal root ganglion degeneration and lower transient receptor potential vanilloid 4 expression,^9^ and GJG may increase peripheral tissue blood flow through nitric oxide-dependent signaling in diabetic rats.^18^ None of these mechanisms has been established in patients, and they should be regarded as hypotheses consistent with, rather than explanations for, the present clinical observation.

The PTX RDI was numerically higher in the GJG arm, although this secondary finding should be interpreted cautiously. Non-hematologic grade 3-5 adverse events were similarly distributed between the 2 arms, suggesting no clear excess of such toxicity attributable to GJG. TTF was similar between the 2 arms, although the estimate was imprecise and did not exclude a modest difference in either direction.

Physician grading also under-recognized CIPN at later courses relative to patient self-report, whereas specificity remained high (Supplementary Data 7). This is consistent with prior reports of discrepancies between clinician-rated and patient-reported CIPN, as clinician assessment incompletely captures patients’ symptom burden.^19^^,^^20^ Because this analysis was not designed to compare grading behavior between the arms, it cannot exclude differential ascertainment; it does, however, support the inclusion of validated patient-reported outcome measures in a future confirmatory trial.

This study has several limitations. First, and most consequentially, the primary endpoint was not independent of clinician behavior. CIPN was graded with CTCAE v5.0 by unblinded treating investigators, and an event was also recorded whenever neuropathy required pharmacologic intervention or led to PTX dose reduction, discontinuation, or cycle delay; these management decisions were left to the treating clinicians without standardized protocol-defined criteria. Knowledge of the allocation could therefore have influenced the recognition and grading of ambiguous symptoms, the thresholds for initiating pharmacologic treatment and for reducing, discontinuing, or delaying PTX, and the timing of endpoint ascertainment. This is a specific risk of ascertainment and management-related bias, distinct from the general limitations of an unblinded design, and it could have operated in either direction. Second, the sample size was calculated for a phase II trial (2-sided α, 0.10; power, 75%), providing exploratory evidence rather than definitive proof of efficacy. Third, the non-lung cancer subgroup comprised only 13 patients (20%), and the point estimate did not indicate a benefit; however, the wide CI and the absence of a significant interaction preclude any firm conclusion regarding differential efficacy based on cancer type. Fourth, as discussed above, the stratified analysis accounting for the minimization factors yielded an attenuated and less precise estimate, so the magnitude and robustness of the treatment effect remain uncertain. Fifth, GJG adherence was not systematically captured in the CRF, and our assessment of tolerability relies on the absence of discontinuation reports to the study secretariat rather than on prospective dose-diary data. Sixth, although the protocol permitted cryotherapy during PTX infusion in both arms, its use was more frequent in the GJG arm (6 of 32 vs 2 of 33) and occurred exclusively at a single institution. In a post hoc sensitivity analysis excluding these 8 patients, the estimate remained in favor of GJG (HR, 0.42; 90% CI, 0.19-0.92; Supplementary Data 3), but this analysis does not resolve the imbalance: the number of exposed patients was small, their distribution between the arms was unequal, and because all use occurred at one center, the effect of cryotherapy cannot be separated from other characteristics of that institution, which was not a minimization factor. Meaningful residual confounding therefore cannot be excluded. Seventh, enrollment extended from April 2022 to September 2025, a period during which variation in concomitant regimens and supportive management over time and between institutions may have introduced clinical heterogeneity in CIPN risk or management; although randomization should have mitigated systematic between-group differences, the small sample size cannot ensure complete balance. Eighth, the multiple comparisons across the secondary, subgroup, course-specific, and sensitivity analyses were not adjusted for multiplicity; the corresponding *P* values should be interpreted descriptively rather than as confirmatory tests.

Despite these limitations, the magnitude and consistency of the treatment effect across the primary and per-protocol analyses support further investigation. Our findings align with prior evidence from patients treated with docetaxel,^8^ preclinical models,^9^ and recent systematic reviews,^5^^,^^6^ supporting a confirmatory trial consistent with the ACTTION recommendations.^21^ Of note, the median starting PTX dose (191.6 mg/m²) was near the standard 200 mg/m², even though a reduced dose of 175 mg/m² was permitted for Asian patients in the IMpower150 trial of platinum- and taxane-based immunochemotherapy.^22^ These findings may apply to patients receiving full-dose PTX-containing regimens. Because 52 of the 65 patients had lung cancer, the present evidence pertains to a predominantly lung-cancer population receiving carboplatin plus PTX-containing chemotherapy, and no claim of efficacy across tumor types is warranted. Future phase III trials should employ a double-blind, placebo-controlled design, include validated patient-reported outcome measures as coprimary or secondary endpoints, and recruit a sufficiently large subset of patients with non-lung cancer to clarify whether the benefit extends across tumor types.

## Conclusion

In this open-label, randomized phase II trial conducted in a predominantly lung-cancer population receiving carboplatin plus PTX, prophylactic GJG was associated with a lower occurrence of grade ≥2 CIPN, without a clear increase in non-hematologic toxicity. The stratified estimate remained in the favorable direction but was attenuated and less precise, and unblinded endpoint ascertainment together with clinician-directed management decisions limits causal certainty. These findings therefore represent a promising phase II preventive signal rather than definitive evidence of efficacy and warrant confirmation in a double-blind, placebo-controlled phase III trial.

## Supplementary Material

oyag352_Supplementary_Data

## Data Availability

The data underlying this article cannot be shared publicly to protect participant privacy. Deidentified individual participant data will not be made available. A study protocol summary based on the CRB-approved protocol is provided as Supplementary Material. Additional methodological materials that are not included in the Supplementary Material may be made available from the corresponding author upon reasonable request, subject to institutional and ethical restrictions.
