# Supplementary material for: Preventive effect of goshajinkigan on paclitaxel-induced peripheral neuropathy: an open-label, randomized phase II study (OLCSG2101)
Source: Oncologist. 2026 Sep 16;31(10):oyag352. doi: 10.1093/oncolo/oyag352 (PMC13612169; doi:10.1093/oncolo/oyag352)
Supplement: Supplementary file 1 [file oyag352_supplementary_data.zip › OLCSG2101_Protocol_Summary.pdf]

Supplementary Material

Study Protocol Summary

Preventive effect of goshajinkigan on paclitaxel-induced peripheral neuropathy: an open-label, randomized phase II study (OLCSG2101)

Protocol summary based on the CRB-approved study protocol

This document summarizes the design, conduct procedures, and analysis plan of the OLCSG2101 trial, as set out in the protocol approved by the Certified Review Board of Okayama University (approval no. CRB21-005; approved 28 September 2021) and its subsequent amendments, most recently Version 2.4 (14 November 2024). It also incorporates the previously published protocol paper (Nakamura N, Makimoto G, Tanaka T, et al. A randomized, open-label phase II study on the preventive effect of goshajinkigan against peripheral neuropathy induced by paclitaxel-containing chemotherapy: the OLCSG2101 study protocol. Respir Investig. 2024;62:897-900). It is provided as Supplementary Material accompanying the manuscript reporting the trial results, submitted to The Oncologist.

Trial registration: Japan Registry of Clinical Trials, jRCTs061210047 (registered 21 October 2021; https://jrct.niph.go.jp/en-latest-detail/jRCTs061210047).

This summary describes the protocol as designed and approved. Where the conducted trial and its final analyses differ in minor operational respects (e.g., final number of participating institutions, enrollment completion date), the main manuscript and its Materials and Methods section are the authoritative source.

1. Trial Identification and Regulatory Oversight

Full title: Preventive effect of goshajinkigan against peripheral neuropathy induced by paclitaxel-containing chemotherapy: an open-label, randomized phase II study.

Acronym: OLCSG2101 (GJG-trial). Sponsoring group: Okayama Lung Cancer Study Group (OLCSG).

Coordinating investigator: Go Makimoto, MD, PhD, Department of Respiratory Medicine, Okayama University Hospital, 2-5-1 Shikata-cho, Kita-ku, Okayama 700-8558, Japan.

Study secretariat: Department of Hematology, Oncology and Respiratory Medicine, Okayama University Graduate School of Medicine, Dentistry and Pharmaceutical Sciences (T. Tanaka, N. Nakamura), and Department of Thoracic Oncology and Medicine, Shikoku Cancer Center (Y. Kato).

Statistician / statistical analysis lead: Isao Oze, Division of Cancer Information and Control, Aichi Cancer Center Research Institute.

Ethics approval: Certified Review Board of Okayama University, approval no. CRB21-005, approved 28 September 2021.

Trial registry: Japan Registry of Clinical Trials, jRCTs061210047, registered 21 October 2021.

Regulatory framework: Conducted as a Specified Clinical Research trial under Japan's Clinical Trials Act, in accordance with the Declaration of Helsinki and Japan's Ethical Guidelines for Medical and Biological Research Involving Human Subjects.

Protocol version summarized here: Version 2.4, dated 14 November 2024.

2. Objective

To determine the prophylactic effect of goshajinkigan (GJG), a traditional Japanese herbal medicine (Kampo), against peripheral neuropathy induced by paclitaxel (PTX)-containing chemotherapy.

3. Study Design

Prospective, two-arm, open-label, randomized, multicenter phase II trial. Patients with a pathologically confirmed malignant tumor scheduled to receive at least four courses of chemotherapy containing carboplatin (CBDCA) plus PTX were randomized 1:1 to a GJG prophylaxis arm (Arm A) or a no-prophylaxis control arm (Arm B).

Primary hypothesis: the incidence of grade ≥2 chemotherapy-induced peripheral neuropathy (CIPN) by the completion of four chemotherapy courses is significantly lower in Arm A than in Arm B. If confirmed, GJG would be judged beneficial for CIPN prevention.

At protocol approval, participation of at least four OLCSG-affiliated institutions was planned; the trial was ultimately conducted at seven institutions in Japan (see manuscript).

4. Eligibility Criteria

Inclusion criteria	Exclusion criteria
1. Age ≥20 years at consent<br>2. ECOG performance status 0–1<br>3. Pathologically confirmed malignant tumor, scheduled for ≥4 courses of CBDCA + PTX (PTX ≥150 mg/m² every 3–4 weeks); any treatment line; concurrent angiogenesis inhibitors (e.g., bevacizumab) or immune checkpoint inhibitors (e.g., atezolizumab, pembrolizumab, nivolumab) permitted<br>4. Adequate organ function within 14 days before registration (leukocytes ≥3,000/µL and neutrophils ≥1,500/µL; hemoglobin ≥9.0 g/dL; platelets ≥100,000/µL; AST/ALT ≤100 IU/L; total bilirubin ≤1.5 mg/dL; creatinine ≤2.0 mg/dL)<br>5. Written informed consent from the patient	1. Prior taxane exposure (PTX, albumin-bound PTX, docetaxel, or cabazitaxel)<br>2. History of CIPN<br>3. Current use of duloxetine, pregabalin, mirogabalin, gabapentin, mecobalamin, vaccinia virus-inoculated rabbit skin inflammatory extract, tricyclic antidepressants, or GJG (including over-the-counter forms)<br>4. Overt peripheral neuropathy of any cause (e.g., orthopedic disease, diabetes, prior treatment-related neuropathy)<br>5. Inability to take GJG orally<br>6. Pregnant, possibly pregnant, or breastfeeding<br>7. Any other reason judged unsuitable by the investigator/sub-investigator

5. Randomization

After eligibility confirmation, registration information was submitted to the study secretariat by password-protected email. Allocation was then performed using INDICE Cloud, a web-based patient registration and randomization system, with minimization by cancer type and age. The allocation result was communicated to each site by email.

6. Interventions

6.1 Arm A: GJG prophylaxis

Oral GJG (TJ-107; Tsumura & Co., Tokyo, Japan) 7.5 g/day (3 sachets), divided into 2–3 doses, starting the morning of day 1 of the first CBDCA + PTX course and continuing until at least 21 days after the fourth course (day 22). Timing relative to meals was not restricted and could be changed during the trial. Eligibility and treatment were not restricted by the patient's traditional Kampo constitutional assessment (“Sho”).

6.2 Arm B: No prophylaxis (standard care)

No prophylactic treatment for CIPN. Once grade ≥2 CIPN developed, symptomatic treatment (including GJG) could be started per standard clinical practice.

6.3 Concomitant treatment

Permitted in both arms: limb cryotherapy and compression therapy (surgical gloves) during PTX infusion, preferably starting from course 1; granulocyte colony-stimulating factor; systemic corticosteroids; concurrent radiotherapy (weekly PTX regimens were not eligible); analgesics or opioids for non-CIPN

pain.

Prohibited, except after a grade ≥2 CIPN event: duloxetine, pregabalin, mirogabalin, gabapentin, mecobalamin, vaccinia virus-inoculated rabbit skin inflammatory extract, tricyclic antidepressants, and over-the-counter GJG.

6.4 Protocol-treatment discontinuation criteria

Protocol treatment (GJG prophylaxis in Arm A) was discontinued if any of the following occurred: (1) grade ≥2 CIPN ("event"; TTF follow-up continues); (2) discontinuation of CBDCA + PTX because of disease progression or adverse events; (3) PTX dose reduced below 150 mg/m² per administration; (4) a GJG-related adverse event precluding continuation; (5) an intercurrent illness or complication precluding continuation; (6) withdrawal of consent; (7) post-registration ineligibility; (8) termination of the overall trial; (9) new safety information regarding GJG; (10) investigator judgment that continuation was not feasible.

7. Endpoints

7.1 Primary endpoint

The primary endpoint was the occurrence of grade ≥2 CIPN by completion of four chemotherapy courses. The prespecified primary comparison evaluated time to first grade ≥2 CIPN using Kaplan–Meier methods and a two-sided log-rank test at $\alpha=0.10$, with Cox regression used to estimate the hazard ratio.

7.2 Secondary endpoints

1. Time from chemotherapy initiation to onset of grade ≥2 CIPN
2. CIPN incidence and severity at the end of each course (CTCAE grade and Patient Neurotoxicity Questionnaire [PNQ])
3. Relative PTX dose intensity
4. Adverse events other than peripheral neuropathy
5. Proportion with grade ≥2 CIPN by course 4, excluding patients who discontinued before course 4 for reasons other than CIPN
6. Time to treatment failure (TTF) — added as a secondary endpoint by protocol amendment registered on the Japan Registry of Clinical Trials on 19 January 2023, during the enrollment period

7.3 CIPN assessment and event definition

CIPN was assessed clinically — new numbness, tingling, or pain in the extremities during chemotherapy — without mandatory objective neurological or neurophysiological testing, and was attributed to chemotherapy when no other cause (e.g., diabetes, uremia, vitamin B1 deficiency, neuromuscular or orthopedic disease) was evident.

Peripheral motor and peripheral sensory neuropathy were graded per CTCAE version 5.0; autonomic

neuropathy was not assessed, and motor and sensory components were not distinguished. Acute, transient taxane-associated pain syndromes resolving within 5–6 days were excluded; only cumulative (chronic) CIPN was evaluated.

An "event" was defined as grade ≥2 CIPN. This was also operationalized to include any case in which CIPN necessitated a therapeutic intervention or led to PTX dose reduction, discontinuation, or interval extension. When establishing the event date, the patient's care diary or recollection took priority over the date of physician documentation.

7.4 Time to treatment failure (TTF) — key definitional points

Not applicable to patients receiving the regimen as postoperative adjuvant chemotherapy. Calculated from the randomization date to the earliest of: discontinuation of CBDCA + PTX (or its maintenance therapy) for any reason (disease progression, treatment toxicity, or patient preference), confirmed disease progression, or death.

The protocol specifies detailed operational rules for the discontinuation date (the date discontinuation was decided, not the date maintenance therapy began), ascertainment of disease progression (clinical or radiologic; RECIST assessment not mandatory), planned chemotherapy holidays (not counted as failure unless progression occurs during the holiday), and second primary cancers (do not end or censor TTF follow-up). Patients still on treatment or progression-free at last contact were censored at the last confirmed survival or progression-free date.

8. Sample Size Calculation

The sample size was based on a prior open-label randomized trial comparing GJG with vitamin B12 for the prevention of docetaxel-induced CIPN in breast cancer, which reported grade ≥2 CIPN incidences of 18.2% (GJG) and 48.1% (vitamin B12) (Abe H, et al. Asian Pac J Cancer Prev. 2013;14:6351–6).

Based on these data, incidences of 20% (GJG arm) and 50% (control arm) were assumed for this trial. With a two-sided α of 0.10 and statistical power of 75%, 27 patients per arm (54 total) were required. Anticipating approximately 20% attrition for reasons unrelated to CIPN — based on the completion rate of four PTX courses in a first-line CBDCA + PTX + pembrolizumab trial in squamous NSCLC (Paz-Ares L, et al. N Engl J Med. 2018;379:2040–51) — the target enrollment was set at 33 patients per arm (66 total).

9. Statistical Analysis Plan

9.1 Analysis populations

Efficacy analyses: all randomized patients who did not withdraw consent (full analysis set / intention-to-treat population), analyzed according to allocated treatment regardless of treatment actually received.

Safety analyses: all patients who received at least one dose of the allocated treatment — i.e., ≥1 GJG dose plus ≥1 chemotherapy course in Arm A, or ≥1 chemotherapy course (with or without symptomatic treatment) in Arm B.

9.2 Primary analysis

The cumulative incidence of grade ≥2 CIPN was estimated by the Kaplan–Meier method and compared between arms with a two-sided log-rank test at an overall significance level of 0.10; no adjustment for multiplicity was applied. Cox proportional-hazards regression was used to estimate the hazard ratio.

9.3 Subgroup analyses

Prespecified exploratory subgroup analyses, among all eligible patients, by the randomization (minimization) factors: cancer type (lung vs. non-lung) and age (<70 vs. ≥70 years).

9.4 Safety analysis

Descriptive summary of the number and proportion of patients in each arm with CTCAE grade 1–5 adverse events.

9.5 Other analytical provisions

No formal interim analysis or stopping rule was planned. Extreme or outlying continuous values were retained in the analysis rather than excluded. If the PNQ questionnaire or patient care diary could not be collected, CIPN severity could be assessed from medical-record documentation, telephone contact, or a later in-person interview; when no such information could be obtained (e.g., because of death, impaired consciousness, or transfer of care), the relevant item was treated as missing and, for the primary endpoint, censored.

10. Protocol Amendments

The addition of time to treatment failure (TTF) as a secondary endpoint was registered with the Japan Registry of Clinical Trials on 19 January 2023, during the patient-enrollment period, and is reflected in the endpoints and analysis plan above. Administrative amendments (e.g., changes to trial-office contact details) are recorded in the version history maintained by the Certified Review Board of Okayama University and are not detailed in this summary.

11. Informed Consent and Ethical Conduct

Written informed consent was obtained from every participant by the investigator or sub-investigator, using a patient information and consent form approved by the Certified Review Board, before any screening or study procedure. Surrogate consent and assent procedures were not applicable to this adult trial population.

Participants were free to decline participation or withdraw consent at any time without disadvantage to their subsequent care. If new information relevant to a participant's continued participation became available, or the protocol or consent form was revised in a way that could affect participants' willingness to continue, participants were to be informed and re-consent obtained using the Certified Review Board-approved revised documents.

The trial was conducted in accordance with the Declaration of Helsinki, Japan's Clinical Trials Act, and

Japan's Ethical Guidelines for Medical and Biological Research Involving Human Subjects.

12. Data Confidentiality and Retention

Each participant was assigned a study-specific identification code; a separate correspondence list linking identity to code was maintained by the investigator and kept password-protected. Source data were de-identified (name removed) before use in the study database. Publications and public disclosures do not include directly identifying information (e.g., name, date of birth).

Essential trial documents (protocol, consent forms, case report forms, monitoring records, Certified Review Board correspondence, etc.) and study data are retained for five years after study completion at each institution, in a locked location, and are then destroyed with appropriate safeguards for personal information (secure deletion of electronic data; shredding of paper records).

13. Safety Reporting, Monitoring, and Compensation

Investigators reported serious adverse events to the coordinating investigator and, as required under Japan's Clinical Trials Act, to the Certified Review Board and, for specified reportable events, to the Ministry of Health, Labour and Welfare, within statutory timeframes (7 days for unexpected deaths or life-threatening events; 15 days for other reportable serious adverse events).

On-site and central monitoring were conducted according to a separate monitoring plan; an independent audit was not performed.

Participants were covered by clinical trial insurance, which could provide compensation for medical costs and, in the event of serious and unexpected trial-related health impairment (including death or grade 1–2 permanent disability), further compensation, in accordance with the trial's insurance terms.

14. Trial Organization and Funding

Trial group: Okayama Lung Cancer Study Group (OLCSG). Funding for the trial and the reported analysis is described in the Study Funding section of the main manuscript. The original protocol document stated that the study would be conducted using institutional/departmental research funds.

Conflict-of-interest disclosures applicable to the published trial results are provided in the Disclosure of Potential Conflicts of Interest section of the manuscript, and supersede the general statement in the original protocol document.

15. Study Period (as Approved)

As approved in Version 2.4: enrollment period through 31 December 2025; overall study period (including analysis) through 30 November 2026. The trial subsequently reached its accrual target and enrollment closed early, in September 2025; final conduct details are reported in the main manuscript's Materials and Methods and Results sections, which take precedence over the planned dates summarized here.

16. References

1. Nakamura N, Makimoto G, Tanaka T, Kato Y, Oze I, Kozuki T, et al. A randomized, open-label phase II study on the preventive effect of goshajinkigan against peripheral neuropathy induced by paclitaxel-containing chemotherapy: the OLCSG2101 study protocol. Respir Investig. 2024;62:897–900. https://doi.org/10.1016/j.resinv.2024.07.017

2. Japan Registry of Clinical Trials. jRCTs061210047. https://jrct.niph.go.jp/en-latest-detail/jRCTs061210047

3. Abe H, Kawai Y, Mori T, Tomida K, Kubota Y, Umeda T, Tani T. The Kampo medicine Goshajinkigan prevents neuropathy in breast cancer patients treated with docetaxel. Asian Pac J Cancer Prev. 2013;14:6351–6356.

4. Paz-Ares L, Luft A, Vicente D, Tafreshi A, Gümüş M, Mazières J, et al. Pembrolizumab plus chemotherapy for squamous non-small-cell lung cancer. N Engl J Med. 2018;379:2040–2051.
