# Supplementary material for: Preventive effect of goshajinkigan on paclitaxel-induced peripheral neuropathy: an open-label, randomized phase II study (OLCSG2101)
Source: Oncologist. 2026 Sep 16;31(10):oyag352. doi: 10.1093/oncolo/oyag352 (PMC13612169; doi:10.1093/oncolo/oyag352)
Supplement: Supplementary file 1 [file oyag352_supplementary_data.zip › GJG_SuppleData_revised_clean.pdf]

Supplementary Data

Supplementary Data 1. Kaplan–Meier curves for the cumulative incidence of grade ≥2 CIPN; per-protocol population

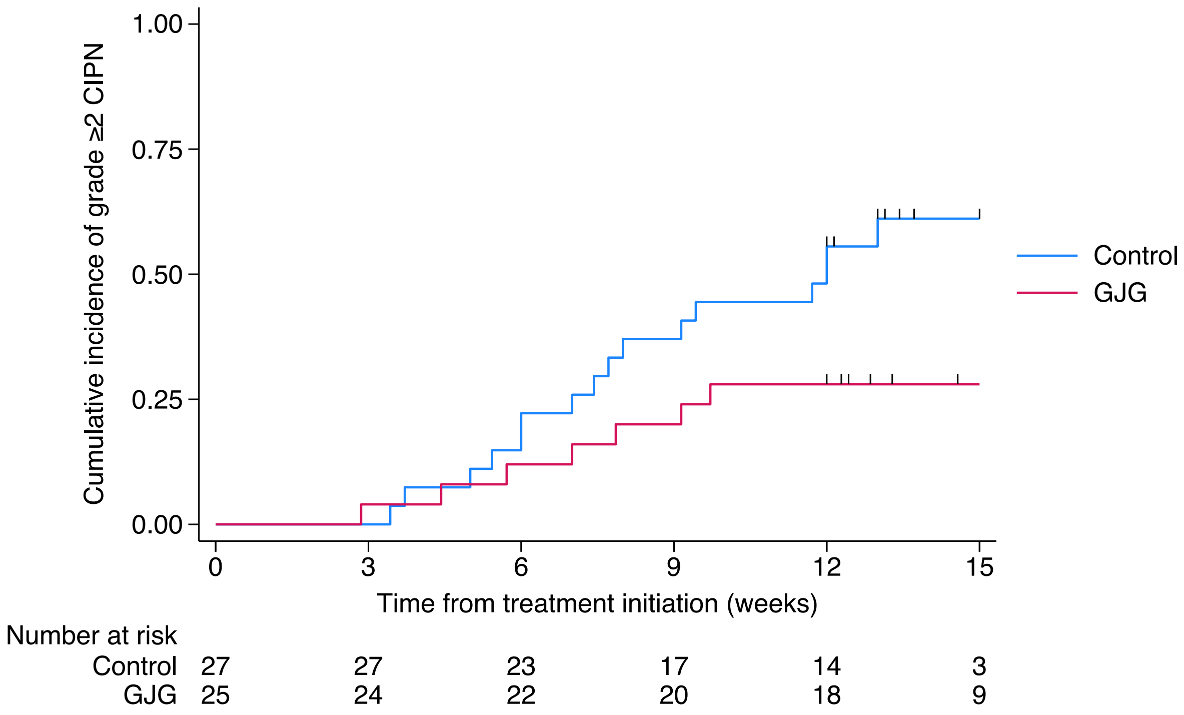

The per-protocol population included patients who completed four chemotherapy courses or discontinued before four courses because of chemotherapy-induced peripheral neuropathy. The x-axis represents time in weeks from treatment initiation and is displayed up to 15 weeks (105 days) for visual clarity, approximately corresponding to the protocol-defined assessment window for the primary endpoint. Numbers at risk are shown below the plot. *P* values are from the log-rank test based on the full follow-up data. This per-protocol analysis is supportive of the protocol-prespecified primary analysis. Confidence intervals for the corresponding effect estimates (Table 2) are presented at the 90% level, corresponding to the two-sided $\alpha = 0.10$ prespecified for the phase II primary endpoint. Abbreviations: CIPN, chemotherapy-induced peripheral neuropathy; GJG, goshajinkigan.

Supplementary Data 2. CIPN incidence and severity by course (ITT, censored at first grade ≥2 event)

	GJG arm				Control arm				*P**
**Course**	**n†**	**G0**	**G1**	**G≥2**	**n†**	**G0**	**G1**	**G≥2**	
1	31	13 (41.9)	16 (51.6)	2 (6.5)	31	17 (54.8)	10 (32.3)	4 (12.9)	0.671
2	26	8 (30.8)	15 (57.7)	3 (11.5)	25	11 (44.0)	8 (32.0)	6 (24.0)	0.291
3	21	6 (28.6)	13 (61.9)	2 (9.5)	17	2 (11.8)	10 (58.8)	5 (29.4)	0.207
4	18	6 (33.3)	10 (55.6)	2 (11.1)	12	2 (16.7)	8 (66.7)	2 (16.7)	1.000

Values are n (%). CIPN graded per CTCAE v5.0. †Patients are censored at first grade ≥2 event; subsequent courses are excluded.

* Fisher's exact test for grade ≥2 vs <2.

Abbreviations: CIPN, chemotherapy-induced peripheral neuropathy; ITT, intention-to-treat; CTCAE, Common Terminology Criteria for Adverse Events; G, grade; GJG, goshajinkigan.

Supplementary Data 3. Post hoc sensitivity analysis excluding patients who received cryotherapy

Population	N	Events (GJG / Control)	HR	90% CI	*P* (log-rank)
Cryotherapy excluded	57	8 / 15	0.42	0.19–0.92	0.061

Eight patients who received limb cryotherapy during paclitaxel infusion (GJG arm, 6 of 32; control arm, 2 of 33), all at a single institution, were excluded from this post hoc sensitivity analysis. The effect estimate remained in favor of GJG; however, because cryotherapy use was uncommon, imbalanced between arms, and confined to one institution, residual confounding by cryotherapy or site cannot be excluded. Confidence intervals are 90%.

Abbreviations: CI, confidence interval; GJG, goshajinkigan; HR, hazard ratio.

Supplementary Data 4. Prespecified exploratory subgroup analysis for grade ≥2 chemotherapy-induced peripheral neuropathy

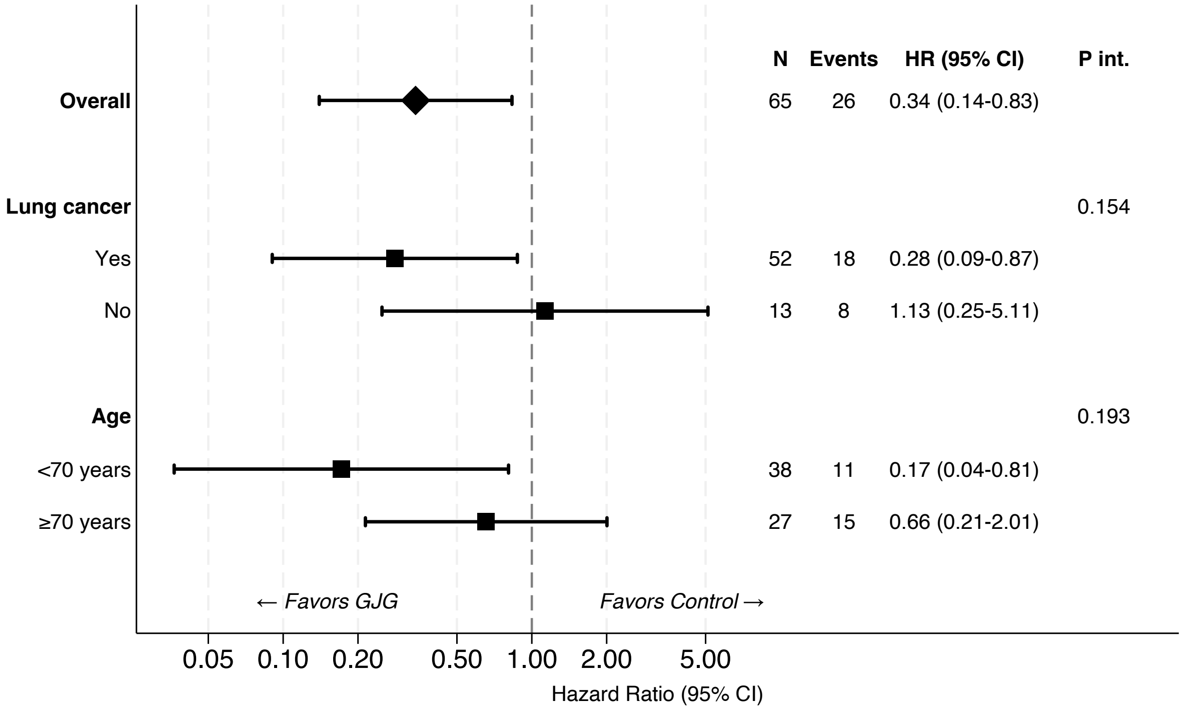

Hazard ratios are presented with 95% CIs because these were prespecified exploratory subgroup analyses. The overall HR is identical to that in Table 2; the CI differs because the primary analysis is reported with a 90% CI. Subgroups were defined by the minimization factors of cancer type and age. Interaction *P* values were not adjusted for multiplicity, and the small subgroup sizes—particularly the 13 patients with non-lung malignancies—preclude firm conclusions.

Abbreviations: CIPN, chemotherapy-induced peripheral neuropathy; CI, confidence interval; GJG, goshajinkigan; HR, hazard ratio.

Supplementary Data 5. Time to treatment failure (excluding 7 pre-amendment patients)

	N	Events	HR	95% CI	*P* (log-rank)
Control arm (no prophylaxis)	30	27	Reference	—	—
GJG arm (prophylaxis)	28	28	1.16	0.70–1.94	0.559

TTF was added as a secondary endpoint by a protocol amendment registered on January 19, 2023. Seven patients enrolled before the amendment were excluded (N=58). The HR is presented with 95% CIs, and this secondary analysis was not adjusted for multiplicity.

Abbreviations: TTF, time to treatment failure; HR, hazard ratio; CI, confidence interval; GJG, goshajinkigan

.

Supplementary Data 6. Kaplan–Meier curves for time to treatment failure

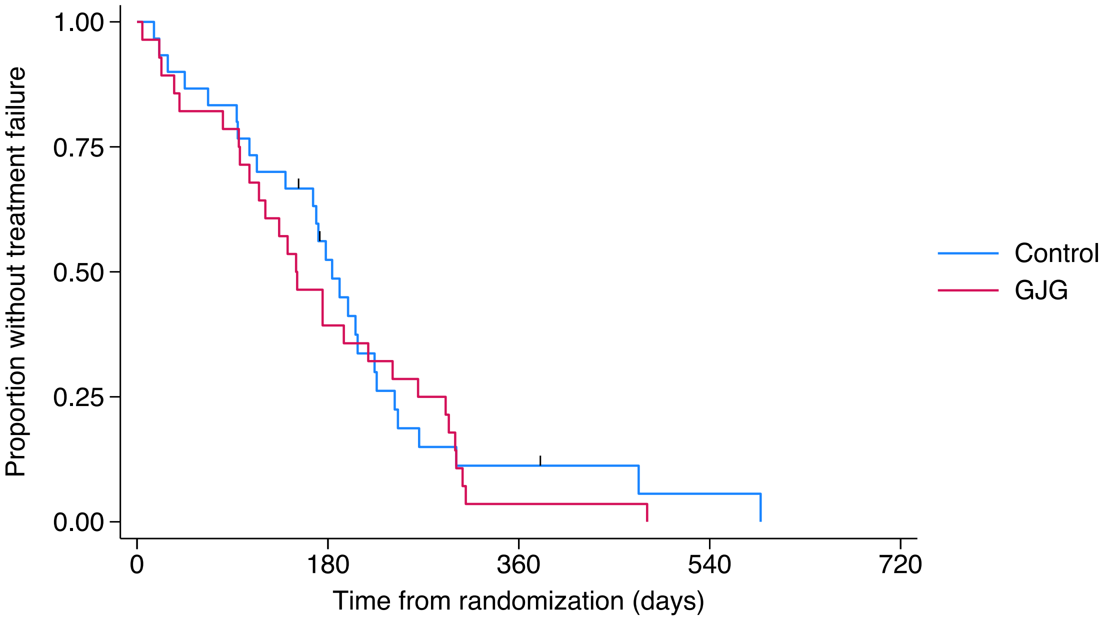

Number at risk					
Control	30	14	3	1	0
GJG	28	11	1	0	0

Time to treatment failure was analyzed after excluding seven patients registered before the protocol amendment that added time to treatment failure as an endpoint. The x-axis represents time in days from randomization. Numbers at risk are shown below the plot. The *P* value is from the log-rank test. Effect estimates with 95% CIs are presented in Supplementary Data 5.

Abbreviations: TTF, time to treatment failure; GJG, goshajinkigan.

Supplementary Data 7. Physician–patient agreement

Panel A. Agreement between physician-rated CIPN grade and patient-reported PNQ (4-level)

Timepoint	N pairs	Exact agreement	κ (quadratic)
Course 1	62	0.726	0.690
Course 2	49	0.735	0.730
Course 3	37	0.676	0.571
Course 4	33	0.576	0.588
Pooled (1–4)	181	0.691	0.673

Quadratic-weighted Cohen κ compared 4-level physician-rated CTCAE grades with patient-reported PNQ categories (A=0, B=1, C=2, D=3). Only paired assessments were included; sample sizes decreased because of treatment discontinuation, missing assessments, and censoring after the first grade ≥2 event.

Abbreviations: CIPN, chemotherapy-induced peripheral neuropathy; CTCAE, Common Terminology Criteria for Adverse Events; PNQ, Patient Neurotoxicity Questionnaire; κ, kappa.

Panel B. Binary agreement at grade ≥2 cutoff: physician vs patient assessment

Timepoint	N pairs	Agreement (%)	Sensitivity (%)	Specificity (%)
Course 1	62	93.5	66.7	96.4
Course 2	49	85.7	75.0	86.7
Course 3	37	78.4	50.0	81.8
Course 4	33	75.8	33.3	91.7
Pooled (1–4)	181	85.1	52.2	89.9

Sensitivity, proportion of patient-reported grade ≥2 correctly identified by physician; Specificity, proportion of patient-reported grade <2 correctly identified by physician.
